# Preliminary Findings on the Effect of Ultrasmall Superparamagnetic Iron Oxide Nanoparticles and Acute Stress on Selected Markers of Oxidative Stress in Normotensive and Hypertensive Rats

**DOI:** 10.3390/antiox11040751

**Published:** 2022-04-09

**Authors:** Lucia Laubertova, Monika Dvorakova, Peter Balis, Angelika Puzserova, Ingrid Zitnanova, Iveta Bernatova

**Affiliations:** 1 Faculty of Medicine, Institute of Medical Chemistry, Biochemistry and Clinical Biochemistry, Comenius University, Sasinkova 2, 813 72 Bratislava, Slovakia; lucia.laubertova@fmed.uniba.sk (L.L.); ingrid.zitnanova@fmed.uniba.sk (I.Z.); 2 Institute of Normal and Pathological Physiology, Centre of Experimental Medicine, Slovak Academy of Sciences, v.v.i., 813 71 Bratislava, Slovakia; peter.balis@savba.sk (P.B.); angelika.puzserova@savba.sk (A.P.); iveta.bernatova@savba.sk (I.B.)

**Keywords:** acute stress, erythrocytes, hypertension, oxidative stress, markers of oxidative damage, ultrasmall superparamagnetic iron oxide nanoparticles, plasma, USPIONs

## Abstract

Several studies have reported that the administration of various nanoparticles in vivo can cause oxidative stress. The combination of ultrasmall superparamagnetic iron oxide nanoparticles (USPIONs) and acute stress was selected because, during intravenous application of a contrast agent, patients are exposed to psycho-emotional stress. This study was designed to investigate the effect of acute stress and USPIONs on selected markers of oxidative stress (antioxidant capacity, superoxide dismutase, glutathione peroxidase and catalase activities, levels of advanced oxidation protein products, protein carbonyls, lipoperoxides and 8-isoprostanes) in plasma and erythrocytes in normotensive Wistar–Kyoto rats (WKY) and spontaneously hypertensive rats (SHR). In the WKY and SHR groups, there was a significant main effect of genotype between groups on studied markers except protein carbonyls and lipoperoxides. In SHR, the combination of acute stress and USPIONs increased the antioxidant capacity of plasma and the selected enzyme activities of erythrocytes. In WKY, the combination of acute stress and USPIONs decreased the antioxidant capacity of erythrocytes and reduced levels of advanced oxidation protein products in plasma. Our study points to the fact that, when hypertensive subjects are treated with iron oxide nanoparticles, caution should be taken, especially in stress conditions, since they seem to be more vulnerable to oxidative stress produced by USPIONs.

## 1. Introduction

The enormous development of nanotechnologies in recent years has attracted the attention of biomedical research and has also increased the potential of the use of iron oxide-based magnetic nanoparticles (NPs) in the diagnosis and treatment of many diseases. Biological properties of NPs depend on their size, shape, surface modification, distribution, etc. [1,2,3], where it is important to retain specific physicochemical properties (magnetic susceptibility, surface modification, non-toxicity, good mechanical stability and biocompatibility [4], time interaction, with emphasis on minimal negative interaction with an organism and maximum biostability) [5,6]. With these properties, nanoparticles represent an interesting option for intravenous therapy, diagnostics and magnetic resonance imaging (MRI) [7] and a variety of bioapplications—for example, detection of bacteria or viruses, target-specific drug delivery and magnetic hyperthermia treatment in cancer therapy [8]. A specific group of NPs are ultrasmall superparamagnetic iron oxide nanoparticles (USPIONs), the synthetic particles of maghemite (γ-Fe_2_O_3_) or magnetite (Fe_3_O_4_) with a core size less than 50 nm [9]. In general, uncoated iron oxide-based magnetic nanoparticles can induce DNA breakdown, hemolysis and oxidative stress. Therefore, various coatings have been used to reduce their toxicity and improve their properties [1]. USPIONs are coated with polyethylene glycol (PEG), which ensures their stability and biocompatibility, improves their distribution, and reduces their uptake by the mononuclear phagocytic system [10,11]. The PEG coating of USPIONs reduces undesired interactions with plasma proteins and their subsequent opsonization as well as allowing targeting ligands to be conjugated onto the USPIONs [4,12]. However, using different types of NPs/USPIONs in biomedical applications may cause problems related to their ability to increase oxidative stress and potential toxicity [13,14].

Oxidative stress is often associated with several biochemical, physiological, and pathophysiological actions. The potential ability of nanoparticles to increase oxidative stress is attributed particularly to their ability to contribute to the creation of reactive oxygen species (ROS) [15]. ROS induce oxidative protein modification represented by advanced oxidation protein products (AOPP) and protein carbonyls, peroxidation of fatty acids represented by lipoperoxides and isoprostanes, and other modifications of biomolecules [16,17]. Mammalian cells possess their own antioxidant defense mechanism, which has the ability to eliminate ROS, including superoxide anion and hydrogen peroxide [18]. The antioxidant system of cells involves small molecular antioxidants such as reduced glutathione (GSH) and antioxidant enzymes such as intracellular superoxide dismutase (SOD), glutathione peroxidase (GPx), glutathione S-transferase (GST) and catalase (CAT) [19,20].

Many years of scientific research have shown that redox stress contributes to the development of hypertension [21,22,23,24]. Hypertension is the most common health problem in many countries. Untreated hypertension leads to many cardiovascular diseases, including myocardial infarction, heart failure, stroke, peripheral artery disease as well as chronic kidney disease [25,26]. Growing evidence confirms that oxidative stress plays a crucial role in the pathogenesis of hypertension, but mechanisms contributing to the increased production of ROS in hypertension are still not well understood [27,28]. Since NPs are often used during different diagnostic examinations in hypertensive patients, it is important to study the safety and the different effects of various NPs, especially in conditions of acute psycho-emotional stress, in hypertensive subjects.

To mimic acute stress in rats, we used an “air jet” as a stressor to trigger psycho-emotional stress. This type of stress is characterized by a defense reaction associated with an increase in the activity of the sympathetic nervous system and with an increase in acute blood pressure and heart rate [29,30]. Recently, we showed that air jet-induced stress increased plasma corticosterone levels and reduced USPIONs—originating from iron content in the whole blood and in the liver of WKY [8]. The high level of corticosterone leads to the induction of oxidative stress via the production of excessive ROS [31]. The same model of stress also reduced vascular disorders induced by a single dose of USPIONs in WKY [8]. In addition, it is well known that stress can negatively affect heart function, and may also induce endothelial dysfunction and oxidative stress, which may further modify blood pressure regulation [32,33,34,35,36].

Thus, to study the effects of USPIONs in hypertension, we examined the responses of enzymes involved in antioxidant defense, as well as the markers of oxidative damage in plasma and erythrocytes after a single intravenous infusion of USPIONs in an animal model of hypertension (SHR) and in normotensive rats exposed to acute stress.

## 2. Materials and Methods

### 2.1. Nanoparticles

Ultrasmall superparamagnetic iron oxide nanoparticles coated by polyethylene glycol were used in our study. Commercially available USPIONs (Iron oxide (II, III), magnetic nanoparticles solution, transmission electron microscope determined average particle size 30 nm, PEG functionalized, 1 mg/mL Fe, dispersion in H_2_O, MW: 231.53 g/mol, cat. No. 747408, PubChem SID 329765832, accessed on 7 September 2021) were purchased from Merck, Bratislava, Slovakia (previously Sigma-Aldrich, Bratislava, Slovakia) Before intravenous infusion to rats, USPIONs were autoclaved at 121 °C for 30 min and mixed with sterile saline to reach a final dose of 1 mg of Fe/kg of body weight. Detailed properties of nanoparticles (including their superparamagnetic properties) used in this study were described previously by Skratek et al., 2020 [14].

### 2.2. Animals and Study Design

Rats were born in the certified animal facility of the Institute of Normal and Pathological Physiology, Centre of Experimental Medicine, Slovak Academy of Sciences for a standardized environmental background for all animals. Rats were housed under standard conditions at 22–24 °C in a 12 h light/dark cycle and fed with a pelleted diet Altromin formula 1324, variant P (Altromin Spezialfutter, Lage, Germany), with an iron content of 192.51 mg/kg and tap water ad libitum. Wistar–Kyoto (WKY) and spontaneously hypertensive rats (SHR), 12–16-week-old males, were used in this study. Rats were divided into two groups according to their genotype. Every genotype was divided into four groups: control (C) (WKY_C_, *n* = 6; SHR_C_, *n* = 8), a group exposed to acute stress (S) (WKY_S_, *n* = 6; SHR_S_, *n* = 8), a group exposed to USPIONs (U) (WKY_U_, *n* = 6; SHR_U_, *n* = 6) and a group exposed to the combination of acute stress and USPIONs (S + U) (WKY_S+U_, *n* = 5; SHR_S+U_, *n* = 6).

The day before the experiment (approximately 20–24 h), two catheters were implanted into animals under anesthesia (2.5–3.5%; isoflurane, FORANE, 99.9% liquid for steam for inhalation, b.n. 6065955, AbbVie s.r.o., Bratislava, Slovak Republic). One catheter was implanted into the carotid artery for direct blood pressure measurement and the second catheter into the jugular vein for drug (saline/USPIONs) delivery, as described previously by Liskova et al. 2020 [8]. All rats were also pre-treated with meloxicam (Meloxidolor, Le Vet Beheer B.V., Oudewater, Nederland) at 2 mg/kg of body weight, intramuscularly, before surgery to prevent post-surgical pain.

**Figure 1:**
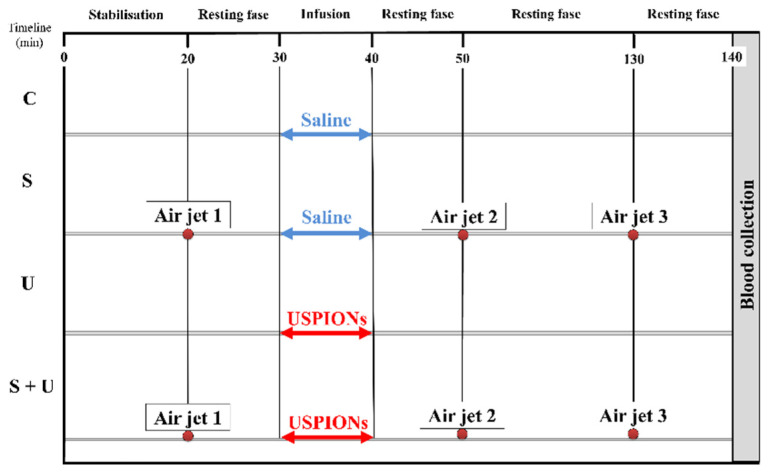
Timeline of the experimental protocol. Acute stress was produced by a 5 s pulse of air to the forehead of the rat, as described previously [8]. C—control; S—acute stress; U—USPIONs; S + U—the combination of acute stress and USPIONs.

During the experiments, the conscious rats were placed into a plastic box with dark walls and a transparent lid (27 cm × 14 cm × 9 cm in size), which allowed the rats free movement. Rats in controls were treated with 10 min infusions of saline, starting approximately 30 min from the beginning of the experiment (Figure 1). Acute stress in rats was induced by a 5 s pulse of air using an air jet 10 min before and 10 and 90 min after USPIONs or saline administration, as described in detail by Liskova et al. 2020 [8]. Rats exposed to USPIONs were treated with 10 min infusions of USPIONs at a dose of 1 mg Fe/kg of body weight. Saline was administered to control rats and rats exposed to stress alone.

### 2.3. Sample Preparation

Arterial blood was collected into commercial K3 EDTA (tripotassium ethylenediaminetetraacetic acid) coated tubes (FL Medical, Padova, Italy). Samples were centrifuged (665× *g*, 10 min, 4 °C, to minimize the damage to erythrocytes used for other measurements) and plasma was aliquoted, shock frozen, and stored at −80 °C until analysis. Blood plasma aliquots were used for determination of antioxidant capacity, protein carbonyls, lipoperoxides and 8-isoprostanes.

For isolation of erythrocytes, sediment of blood was used. Erythrocytes were washed three times with physiological solution. After the final centrifugation (665× *g*, 5 min, 4 °C), erythrocytes were hemolyzed in chilled distilled water. Lysates of erythrocytes were aliquoted, frozen, and stored at −80 °C until analysis [37]. They were used for determination of the antioxidant capacity and the activities of antioxidant enzymes (superoxide dismutase, glutathione peroxidase and catalase). Before analysis, the concentration of proteins in samples (plasma and lysates of erythrocytes) was measured by the Micro BCA™ Protein Assay Kit (Thermo Fisher Scientific Inc., Waltham, MA, USA, cat. No. 23235) according to the manufacturer’s protocol.

### 2.4. Measurement of Antioxidant Capacity

Antioxidant capacity was determined in plasma and lysates of erythrocytes by the Trolox Equivalent Antioxidant Capacity (TEAC) method with minor modifications [38]. This assay assesses total radical scavenging capacity based on the ability of a compound to scavenge the stable 2,2′-azino-bis(-3-ethylbenzothiazoline-6-sulfonic acid radical cation (ABTS⁺). Addition of antioxidants reduces the ABTS⁺ radical cation to an extent depending on the activity and the duration of the reaction. Antioxidant capacity was measured at 734 nm and room temperature. Antioxidant capacity is expressed in mmol of trolox/L/mg of proteins using trolox (a hydrophilic form of vitamin E) as a standard.

The blue-green radical cation was generated by oxidation of 2,2′-azino-bis(-3-ethylbenzothiazoline-6-sulfonic acid) (ABTS; 7 mmol/L; Merck, Sigma-Aldrich, Taufkirchen, Germany) with potassium persulfate (2.45 mmol/L; Merck, Sigma-Aldrich, Taufkirchen, Germany) in water. This solution was stored in the dark at room temperature for 12–16 h before use. Stock solutions of trolox (2.5 mmol/L; Merck, Sigma-Aldrich, Taufkirchen, Germany) were prepared in phosphate-buffered saline (PBS; 10 mmol/L; pH 7.4, Merck, Sigma-Aldrich, Taufkirchen, Germany) and then diluted at a concentration range of 0.05–0.5 mmol/L for use as a working standard. Fresh working standards were prepared daily.

To prepare the ABTS⁺ working solution, the concentrated ABTS▪+ solution was diluted with water to a final absorbance of 0.70 ± 0.02 at 734 nm at room temperature. To the microplate, 10 μL standards, plasma samples or lysates of erythrocytes were added to 290 μL ABTS⁺ solution and the absorbance at 734 nm was measured over time. This was compared to a blank, where 10 μL of water was added to 290 μL of the ABTS▪+ solution. The reduction in absorbance was determined 10 min after addition of the samples. The TEAC of the samples was calculated by relating this decrease in absorbance to that of a trolox solution on a molar basis.

### 2.5. Measurements of Antioxidant Enzymes

Superoxide dismutase activity was determined in lysates of erythrocytes by the Superoxide dismutase determination kit (Merck, Sigma-Aldrich, Taufkirchen, Germany, cat. No. 19160-1KT-F) according to the manufacturer’s protocol. SOD activity was expressed in U/mg of proteins.

Glutathione peroxidase activity was determined in lysates of erythrocytes by the Glutathione peroxidase assay kit (Cayman Chemical Company, Ann Arbor, MI, USA, cat. No. 703102) according to the manufacturer’s protocol. GPx activity was expressed in U/mg of proteins.

Catalase activity was determined in lysates of erythrocytes by the catalase assay kit (Cayman Chemical Company, Ann Arbor, MI, USA, cat. No. 707002) according to the manufacturer’s protocol. CAT activity was expressed in U/mg of proteins.

### 2.6. Measurements of Parameters of Oxidative Damage to Proteins

The level of advanced oxidation protein products in plasma was measured by the modified method according to Kalousova et al. (2002) [39].The exact AOPP structures are unknown. For their determination, the wavelength of their maximum of 340 nm is used. Chloramine T has the same absorption maximum after the reaction with KI and is used as the standard. AOPP levels are expressed in nmol/mg of plasma proteins.

The day before determination of AOPP, 30 μL of precipitating agent was added to 150 μL of blood plasma and allowed to precipitate lipids overnight at 4 °C. The precipitating agent was prepared by mixing MgCl_2_. 6H_2_O (1 mol/L; Merck; Sigma-Aldrich, Taufkirchen, Germany) and dextran sulphate (2%; Merck, Sigma-Aldrich, Taufkirchen, Germany) at a ratio of 1:1 before use. Precipitated lipids were removed by centrifugation at 1000× *g* for 20 min at room temperature. The supernatant was appropriately diluted at a ratio of 10:1 with PBS (10 mmol/L; pH 7.4). To the microplate, 100 μL of diluted samples and 10 μL of glacial acetic acid (Merck, Sigma-Aldrich, Taufkirchen, Germany) were applied.

A stock solution of chloramine T (10 mmol/L, Merck, Sigma-Aldrich, Taufkirchen, Germany) was prepared in PBS (10 mmol/L, pH 7.4, Merck, Sigma-Aldrich, Taufkirchen, Germany) and then diluted at a concentration range of 10–100 μmol/L for use as a working standard. Fresh working standards were prepared daily. For calibration, 100 μL of chloramine T (10–100 μmol/L) and 100 μL of PBS as a blank were applied to a microplate. To each standard, 5 μL of KI (1.16 mol/L; Merck, Sigma-Aldrich, Taufkirchen, Germany) and 10 μL of glacial acetic acid were added and absorbance at 340 nm was measured.

The level of protein carbonyls in plasma was measured by the Oxiselect^TM^ protein carbonyl ELISA kit (Cell biolabs, Inc., San Diego, CA, USA, cat. No. STA-310) according to the manufacturer’s protocol. Protein carbonyl levels are expressed in nmol/mg of plasma proteins.

### 2.7. Measurements of Parameters of Oxidative Damage to Lipids

The level of lipoperoxides in plasma was measured by the modified method according to el-Saadani et al. (1989) [40].

The analysis was based on the ability of lipoperoxides to convert iodide to iodine. The iodine in the reaction mixture gradually reacted with an excess of iodide to form a triiodide anion with an absorption maximum of 365 nm. Lipoperoxide levels are expressed in nmol/mg of plasma proteins.

For determination of lipoperoxide levels, 20 μL of plasma sample and 200 μL of working solution were added to the microplate. The working solution consisted of potassium dihydrogenphosphate (0.2 mol/L; Merck, Sigma-Aldrich, Taufkirchen, Germany), potassium iodide (0.12 mol/L, Merck, Sigma-Aldrich, Taufkirchen, Germany), sodium azide (0.15 mmol/L; Merck, Sigma-Aldrich, Taufkirchen, Germany), Igepal (3 mmol/L, Merck, Sigma-Aldrich, Sigma-Aldrich, Taufkirchen, Germany), benzalkonium chloride (0.27 mmol/L; Merck, Sigma-Aldrich, Taufkirchen, Germany), and ammonium molybdate (10 μmol/L; Merck, Sigma-Aldrich, Taufkirchen, Germany). Lipoperoxide levels were calculated according to Lambert–Beer law using the molar absorption coefficient of triiodide anion (ε = 24,600 L.mol^−1^.cm^−1^) measured at 365 nm.

The level of plasma 8-isoprostanes (Iso-P) was determined by the 8-isoprostanes express ELISA kit (Cayman Chemical Company, Ann Arbor, MI, USA, cat. No. 516360) according to the manufacturer’s protocol. 8-Isoprostanes levels are expressed in pg/mg of plasma proteins.

### 2.8. Statistical Analysis

The statistical analyses were performed using statistical software StatsDirect 3, version 3.2.109. (StatsDirect^®^ Ltd., Birkenhead, UK), and GraphPad Prism, version 5.0 (GraphPad Software, Inc., San Diego, CA, USA). The level of significance was defined as *p* < 0.05. The data are presented as the mean ± standard error of the mean (SEM). Normality of data was analyzed by the Shapiro-Wilk test. Analysis of variance (ANOVA) was used on the normally distributed data. Differences between controls of each genotype were analyzed by one-way ANOVA with Bonferroni comparison. Differences between groups of each genotype were analyzed by two-way ANOVA with Bonferroni multiple comparison. To detect associations between genotypic groups, two-way ANOVA with Bonferroni comparison was used. The associations between parameters were analyzed with Pearson’s correlations.

## 3. Results

### 3.1. The Antioxidant Capacity of Plasma

**Figure 2:**
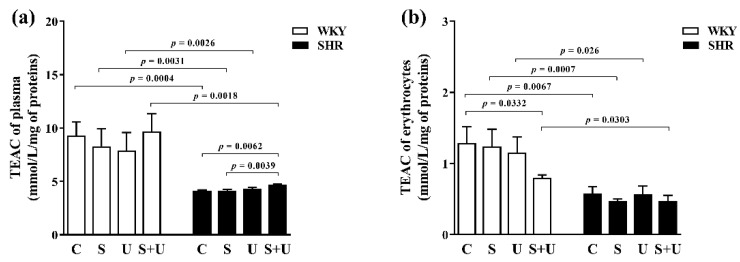
Effects of USPIONs and acute stress on the antioxidant capacity of plasma (**a**) and erythrocytes (**b**) in normotensive (WKY) and spontaneously hypertensive rats (SHR). The level of significance was defined as *p* < 0.05. The data are presented as the mean ± standard error of the mean (SEM). TEAC—Trolox Equivalent Antioxidant Capacity; WKY-Wistar—Kyoto rats; SHR—spontaneously hypertensive rats; C—control; S—acute stress; U—USPIONs; S + U—the combination of acute stress and USPIONs.

In WKY, there were no significant changes in the antioxidant capacity of plasma between groups (Figure 2a). Acute stress, USPIONs and the combination of acute stress and USPIONs had no effect on the antioxidant capacity of plasma of WKY.

In plasma of SHR (Figure 2a), the SHR_S+U_ group had significantly higher antioxidant capacity compared to the control SHR_C_ group (SHR_S+U_ vs. SHR_C_, *p* = 0.0062) and compared to SHR exposed to acute stress only (SHR_S+U_ vs. SHR_S_, *p* = 0.0039). Acute stress and USPIONs, respectively, had no effect on the antioxidant capacity of plasma of SHR.

A significant reduction in plasma antioxidant capacity was found in the SHR groups compared to the WKY groups with the same exposure in each pair, including the control groups (SHR_C_ vs. WKY_C_, *p* = 0.0004; SHR_S_ vs. WKY_S_, *p* = 0.0031; SHR_U_ vs. WKY_U_, *p* = 0.0026; SHR_S+U_ vs. WKY_S+U_, *p* = 0.0018) (Figure 2a).

In genotype groups of WKY and SHR, there was a statistically significant association for the antioxidant capacity of plasma between groups (main effect of genotype, F_(1,43)_ = 39.78, *p* < 0.0001, *n* = 51).

### 3.2. The Antioxidant Capacity of Erythrocytes

In WKY, acute stress and USPIONs had no significant effect on the antioxidant capacity of erythrocytes (Figure 2b). The WKY_S+U_ group had significantly reduced antioxidant capacity of erythrocytes compared to the control WKY_C_ group (WKY_S+U_ vs. WKY_C_, *p* = 0.0332).

In SHR, there were no significant changes in the antioxidant capacity of erythrocytes between groups. Acute stress, USPIONs and the combination of acute stress and USPIONs had no significant effect on the antioxidant capacity of erythrocytes in SHR (Figure 2b).

In the control groups of different genotypes, significant changes in the antioxidant capacity of erythrocytes were found. The control SHR_C_ group had significantly reduced antioxidant capacity of erythrocytes compared to the control WKY_C_ group (SHR_C_ vs. WKY_C_, *p* = 0.0067) (Figure 2b).

A significant reduction in the antioxidant capacity of erythrocytes was found in the SHR groups compared to the WKY groups with the same exposure (SHR_S_ vs. WKY_S_, *p* = 0.0007; SHR_U_ vs. WKY_U_, *p* = 0.026; SHR_S+U_ vs. WKY_S+U_, *p* = 0.0303) (Figure 2b).

In genotype groups of WKY and SHR, there was a statistically significant association for the antioxidant capacity of erythrocytes between groups (main effect of genotype, F_(1,43)_ = 32.25, *p* < 0.0001, *n* = 51).

### 3.3. Superoxide Dismutase Activity

**Figure 3:**
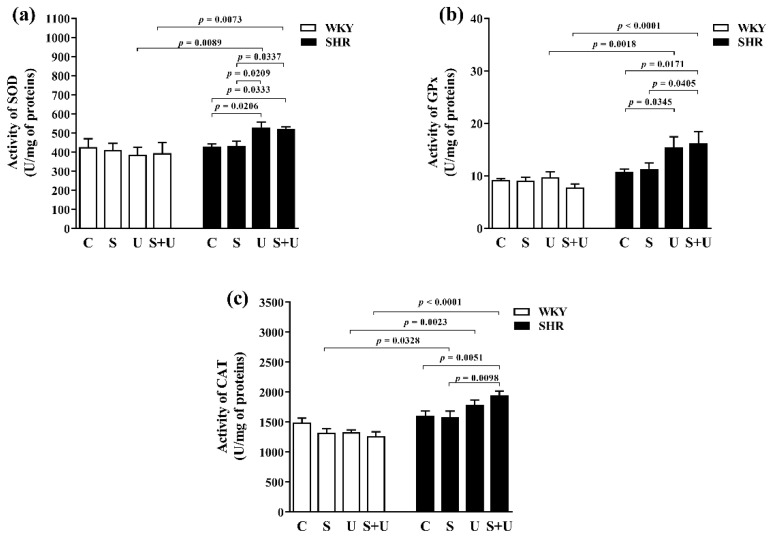
Effects of USPIONs and acute stress on superoxide dismutase (**a**), glutathione peroxidase (**b**) and catalase (**c**) activities in erythrocytes of normotensive (WKY) and spontaneously hypertensive rats (SHR). The level of significance was defined as *p* < 0.05. The data are presented as the mean ± standard error of the mean (SEM). SOD—superoxide dismutase; GPx—glutathione peroxidase; CAT—catalase; WKY-Wistar—Kyoto rats; SHR—spontaneously hypertensive rats; C—control; S—acute stress; U—USPIONs; S + U—the combination of acute stress and USPIONs.

In WKY, there were no significant changes in the superoxide dismutase (SOD) activity between groups (Figure 3a).

In SHR (Figure 3a), significantly increased SOD activity was found in the SHR_U_ group and the SHR_S+U_ group compared to the control SHR_C_ group and the SHR_S_ group (SHR_U_ vs. SHR_C_, *p* = 0.0206; SHR_S+U_ vs. SHR_C_, *p* = 0.0333; SHR_U_ vs. SHR_S_, *p* = 0.0209; SHR_S+U_ vs. SHR_S_, *p* = 0.0337).

A significant increase in SOD activity was found in the SHR groups compared to the WKY groups with the same exposure for groups exposed to USPIONs and the combination of acute stress and USPIONs (SHR_U_ vs. WKY_U_, *p* = 0.0089; SHR_S+U_ vs. WKY_S+U_, *p* = 0.0073) (Figure 3a).

In genotype groups of WKY and SHR, there was a statistically significant association for SOD activity between groups (main effect of genotype, F_(1,43)_ = 9.686, *p* = 0.0033, *n* = 51).

### 3.4. Glutathione Peroxidase Activity

In WKY, there were no significant changes in the glutathione peroxidase (GPx) activity of erythrocytes between groups (Figure 3b).

In SHR (Figure 3b), the SHR_U_ group and the SHR_S+U_ group had significantly higher GPx activity compared to the control SHR_C_ group (SHR_U_ vs. SHR_C_, *p* = 0.0345; SHR_S+U_ vs. SHR_C_, *p* = 0.0171). In addition, the SHR_S+U_ group had significantly higher GPx activity compared to the SHR_S_ group (SHR_S+U_ vs. SHRS, *p* = 0.0405).

There was no significant change in GPx activity in erythrocyte lysates for control groups with different genotypes (Figure 3b).

A significant increase in GPx activity was found in the SHR groups compared to the WKY groups with the same exposure for groups exposed to USPIONs and the combination of acute stress and USPIONs (SHR_U_ vs. WKY_U_, *p* = 0.018; SHR_S+U_ vs. WKY_S+U_, *p* < 0.0001) (Figure 3b).

In genotype groups of WKY and SHR, there was a statistically significant association for GPx activity between groups (main effect of genotype, F_(1,43)_ = 25.90, *p* < 0.0001, *n* = 51).

### 3.5. Catalase Peroxidase Activity

In WKY, there were no significant changes in the catalase (CAT) activity of erythrocytes between groups (Figure 3c).

In SHR (Figure 3c), the SHR_S+U_ group had significantly higher CAT activity compared to the control SHR_C_ group (SHR_S+U_ vs. SHR_C_, *p* = 0.0051) and to the SHR_S_ group (SHR_S+U_ vs. SHR_S_, *p* = 0.0098). Acute stress had no effect on CAT activity compared to the control.

A significant increase in CAT activity was found in the SHR groups compared to the WKY groups with the same exposure for pairs (SHR_S_ vs. WKY_S_, *p* = 0.0328; SHR_U_ vs. WKY_U_, *p* = 0.0023; SHR_S+U_ vs. WKY_S+U_, *p* < 0.0001) (Figure 3c).

In genotype groups of WKY and SHR, there was a statistically significant association for CAT activity between groups (main effect of genotype, F_(1,43)_ = 43.84, *p* < 0.0001, *n* = 51).

### 3.6. Advanced Oxidation Protein Products

The effect of acute stress, USPIONs and the combination of stress and USPIONs on advanced oxidative damage to proteins (AOPP) was studied in terms of advanced oxidation protein product formation.

**Figure 4:**
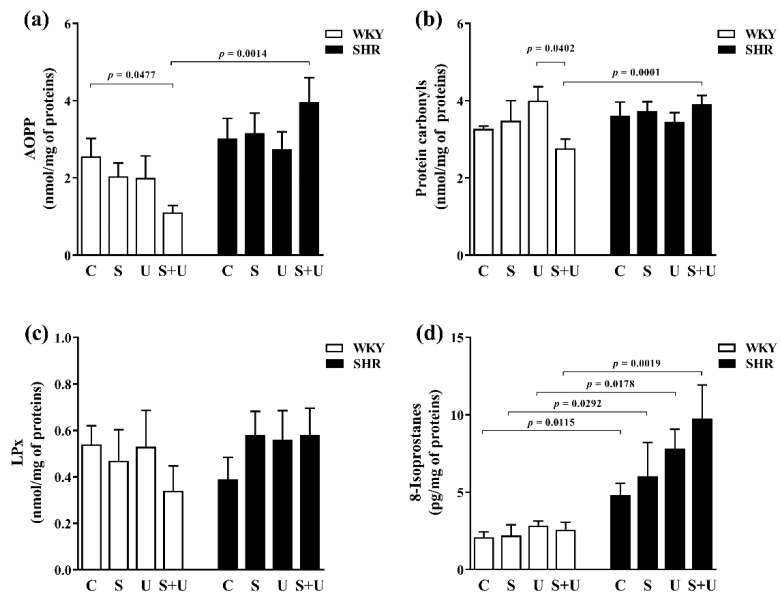
Effects of USPIONs and acute stress on levels of advanced oxidation protein products (**a**), protein carbonyls (**b**), lipoperoxides (**c**), and 8-isoprostanes (**d**) in plasma of normotensive (WKY) and spontaneously hypertensive rats (SHR). The level of significance was defined as *p* < 0.05. The data are presented as the mean ± standard error of the mean (SEM). AOPP—advanced oxidation protein products; LPx—lipoperoxides; WKY-Wistar—Kyoto rats; SHR—spontaneously hypertensive rats; C—control; S—acute stress; U—USPIONs; S + U—the combination of acute stress and USPIONs.

In WKY (Figure 4a), a significant decrease in AOPP levels was found in the WKY_S+U_ group compared to the control WKYC group (WKY_S+U_ vs. WKY_C_, *p* = 0.0477). Acute stress and USPIONs had no significant effect on AOPP formation in plasma of WKY.

In SHR (Figure 4a), there were no significant changes in AOPP levels between groups.

Significant changes in AOPP levels were not found for control groups with different genotypes. The control SHR_C_ group had a slightly higher level of AOPP in plasma compared to the control WKY_C_ group (Figure 4a).

Significantly increased AOPP levels were found in groups exposed to the combination of acute stress and USPIONs only (SHR_S+U_ vs. WKY_S+U_, *p* = 0.0014) for the WKY and SHR groups with the same exposure (Figure 4a).

In genotype groups of WKY and SHR, there was a statistically significant association for AOPP levels between groups (main effect of genotype, F_(1,43)_ = 13.92, *p* = 0.0007, *n* = 51).

### 3.7. Protein Carbonyls

In WKY, acute stress and USPIONs had no significant effect on protein carbonyl formation (Figure 4b). The WKY_S+U_ group had significantly reduced protein carbonyl levels compared to the WKY_U_ group (WKY_S+U_ vs. WKY_U_, *p* = 0.0402).

In SHR (Figure 4b), there were no significant changes in protein carbonyl levels between groups.

There were no significant changes in protein carbonyl levels in plasma for control groups with different genotypes (Figure 4b).

Significantly increased formation of protein carbonyls in plasma was found in the SHR groups compared to the WKY groups exposed to the combination of acute stress and USPIONs (SHR_S+U_ vs. WKY_S+U_, *p* = 0.0163) (Figure 4b).

### 3.8. Lipoperoxides

The effect of stress, USPIONs and the combination of stress and USPIONs on lipid peroxidation was studied in terms of lipoperoxide (LPx) formation.

In WKY and SHR, there were no significant changes in LPx levels between groups (Figure 4c).

There were no significant changes in LPx levels in plasma for control groups as well as groups with the same exposure with different genotypes (Figure 4c).

### 3.9. 8-Isoprostanes

In WKY and SHR, there were no significant changes in the 8-isoprostane (Iso-p) levels between the groups (Figure 4d).

Significant changes in Iso-P levels in plasma were found for control groups with different genotypes. The control SHR_C_ group had significantly higher Iso-P levels compared to the control WKY_C_ group (SHR_C_ vs. WKY_C_, *p* = 0.0115) (Figure 4d).

Significantly increased Iso-P levels were found in the SHR groups compared to the WKY groups with the same exposure for groups exposed to acute stress, USPIONs and the combination of acute stress and USPIONs (SHR_S_ vs. WKY_S_, *p* = 0.0292; SHR_U_ vs. WKY_U_, *p* = 0.0178; SHR_S+U_ vs. WKY_S+U_, *p* = 0.0019) (Figure 4d).

In genotype groups of WKY and SHR, there was a statistically significant association for the Iso-P levels between groups (main effect of genotype, F_(1,43)_ = 28.28, *p* < 0.0001, *n* = 51).

### 3.10. Correlations

In rats with different genotypes, significant correlations between different markers of oxidative stress are presented in Table 1. Correlation is significant at the 0.05 level (2 tailed).

## 4. Discussion

The aim of our study was to investigate an antioxidant response in rats exposed to USPIONs and stress alone, or in combination, in two different rat strains by determination of the antioxidant capacity of plasma and erythrocytes and the activities of antioxidant enzymes in erythrocytes. We also measured levels of markers of oxidative damage to proteins and lipids in plasma.

In hypertension, the production of reactive oxygen species (ROS) is increased by various processes, which can lead to an increase in other cardiovascular diseases and disorders, as shown by many scientific studies [33,35,41,42]. Therefore, the non-enzymatic and enzymatic antioxidant defense is an important mechanism to reduce the risk of cardiovascular diseases and against increased oxidative stress. During diagnostic examinations as well as intravenous application of a contrast agent, patients are exposed to acute stress. Consequently, a psycho-emotional stress response is induced, and this can affect physiological and biochemical processes in the body [43]. In addition, psycho-emotional stress leads to the induction of various negative effects on different cell types [31].

In our study, we found significantly reduced antioxidant capacity in plasma of young SHR compared to WKY. Similarly, Newaz et al. (1999) demonstrated a significant reduction in total antioxidant status in plasma of young SHR compared to WKY of the same age [44]. In addition, in WKY, we have found a negative correlation between plasma antioxidant capacity and markers of oxidative damage to proteins (AOPP).

Acute stress and USPIONs showed genotype-dependent effects on the antioxidant capacity of plasma. In plasma of WKY, none of the exposures had any effect on antioxidant capacity. On the other hand, USPIONs significantly increased the antioxidant capacity of plasma of SHR that were simultaneously exposed to the combined effect of USPIONs and stress, compared to the control and stress groups. Increased antioxidant capacity could be caused by a combination of the effects of various molecules—for example, by polyethylene glycol (PEG), which is used in nanoparticles. Juarez-Moreno et al. (2015) reported that PEG has the ability to increase antioxidant capacity because it might function as an antioxidant molecule [45]. The same effect could be caused by other molecules such as albumin, bilirubin or uric acid [16]. Because antioxidant capacity in the WKY group was significantly higher than in the SHR group, this final effect might not be visible.

The study of the effects of USPIONs on erythrocytes is challenging. Erythrocytes are very sensitive to variation in their environment and reflect those changes [46]. In the SHR group, significantly reduced antioxidant capacity of erythrocytes was found compared to the WKY group. There was no other effect of the monitored factors. In the study by Radosinska et al. (2021), significantly lower amounts of iron originating from USPIONs were found in erythrocytes of SHR than of WKY using SQUID magnetometry [47]. Decreased uptake of USPIONs into erythrocytes of SHR could be one of the reasons why USPIONs had no effect in SHR animals [47]. Oleksa et al. (2021) reported that administration of USPIONs had no effect on the fundamental properties and deformability of erythrocytes in SHR [12]. In the WKY group, acute stress and USPIONs significantly reduced antioxidant capacity in erythrocytes. A slight reduction trend in antioxidant capacity was observed for all WKY exposure groups; as such, it is possible that only the combination of acute stress and USPIONs caused a significant reduction effect in antioxidant capacity. The study by Oleksa et al. (2021) demonstrated changes in vascular function in WKY induced by USPIONs but no changes in SHR [12].

Iron plays a key role in living matter as a mediator of many important biochemical reactions due to its ability to change oxidation state by providing or accepting electrons. On the other hand, this ability results in the direct or indirect generation of ROS and other free radicals—for example, by the Fenton reaction [48]. Products of this reaction, hydroxyl radicals are extremely reactive and can cause oxidation of basic cellular macromolecules, such as nucleic acids, proteins, lipids, and carbohydrates. Iron plays an important role in the formation of lipid peroxyl radicals and lipoperoxides [20].

Indirect generation of ROS is associated with iron-dependent enzymes that catalyze important biological processes [49]. Erythrocytes eliminate ROS by antioxidant enzymes, such as SOD, GPx and CAT. The overproduction of ROS leads to the increased activities of antioxidant enzymes, as well as increased intracellular levels of these antioxidant enzymes [50].

In the normotensive WKY group, there were no significant changes in the SOD, GPx and CAT activities of erythrocytes between groups. Acute stress, USPIONs and the combination of acute stress and USPIONs had no effect on enzyme activities in erythrocytes. In living systems, the antioxidant defense operates on many levels. Under normal conditions in cells, there is a balance between enzymatic and non-enzymatic antioxidant defense, and elimination of ROS and free radicals. This balance is essential for the survival of organisms and their health [51]. Antioxidant enzymes represent the first level of this defense. Several studies have found that the exposure time to acute stress and nanoparticles is a very important factor in redox balance [4]. Xiong et al. (2015) reported increased SOD activity and reduced oxidative stress in Sprague Dawley rats after iron NPs were administered once per day for 7 days [52]. In the WKY group, defense mechanisms were sufficient to eliminate oxidative processes caused by acute stress, USPIONs or their combination, without changing the activity of antioxidant enzymes. Many research groups have focused on studying the relationship between hypertension and the activities of antioxidant enzymes, but their results were highly contradictory. Although some studies have shown an adaptive increase in the activities of antioxidant enzymes in various models of hypertension, the relationships discovered are still the subject of in-depth discussion [27,53,54,55].

In the SHR group, SOD activity was significant increased after exposure to USPIONs and the combination of acute stress and USPIONs compared to the control group. These two SHR groups also had significantly higher SOD activity compared to the WKY groups with the same exposure. SOD catalyzes the reaction in which hydrogen peroxide (H_2_O_2_) is produced [56]. Subsequently, H_2_O_2_ is decomposed by peroxidases such as GPx or CAT [48]. In this study, a significant increase in GPx and CAT activities in the SHR groups induced by USPIONs or the combination of acute stress and USPIONs was also found. A significant positive correlation between SOD and GPx activities was found in the SHR group [57]. This may indicate an upregulation of GPx activity, possibly caused by the increased production of H_2_O_2_ generated by SOD. Petrulea et al. (2012) reported that increased antioxidant enzyme activities such as SOD, GPx and CAT may be indicators of the compensation of induced oxidative stress [58].

In recent years, a rapid increase in studies focusing on the measurement of oxidatively modified protein and lipid products in the pathogenesis of various diseases has been observed. In the normotensive WKY, levels of markers of oxidative damage to proteins (AOPP and protein carbonyls) were not significantly influenced by stress or USPIONs. However, the combination of these factors resulted in a reduction in levels of measured markers. This indicates a good adaptive response of the internal antioxidant system of the organism to increased stress. In contrast, SHR reacted to increased stress (acute stress + USPIONs) by elevating levels of these markers compared to WKY. Enhanced formation of ROS was previously reported in experimental models of hypertension such as SHR. In addition, increased protein carbonylation was detected in tissues from SHR [59,60]. Tyther et al. (2009) identified 11 proteins including antioxidant enzymes that were differentially carbonylated in SHR kidney medulla compared to WKY normotensive rats [61]. Specific modification of proteins in SHR kidneys may contribute to the renopathy associated with hypertension.

Other consequences of high levels of ROS or free radicals are to inflict direct damage to lipids. Hydroxyl radicals cause oxidative damage to cells because they unspecifically attack biomolecules and they are involved in cellular disorders such as neurodegeneration, cardiovascular diseases, and cancer [17]. Hydroxyl radicals play an important role in the chemistry of lipid peroxidation because they are much stronger oxidants than superoxide anion radicals and can initiate the chain oxidation of various lipids. Hydroxyl radicals are formed through redox cycling by Fenton and Haber–Weiss reactions, where iron plays an important role as a catalyst. In plasma of the two genotype groups of rats (WKY and SHR) in this study, for USPIONs or the combination of acute stress and USPIONs, we found no significant changes in lipoperoxide levels. No changes in this plasma marker could be caused by the decomposition of hydroperoxides by two-electron reduction. Enzymes mainly responsible for this elimination are glutathione peroxidases (GPx) and selenoprotein P. Glutathione peroxidases catalyze the reduction of hydrogen peroxide (H_2_O_2_) and organic hydroperoxides using glutathione (GSH). GPx is both an extracellular and an intracellular enzyme. Selenoprotein P is the major plasma selenoprotein that catalyzes the reduction of phospholipid hydroperoxides using GSH or thioredoxin. This decomposition of lipoperoxides protects plasma proteins against peroxynitrite-induced oxidation and nitration or low-density lipoproteins (LDL) from peroxidation [62].

Isoprostanes are one of the most reliable biomarkers of lipid peroxidation. Isoprostanes are prostaglandin-like compounds produced by non-enzymatic peroxidation of arachidonic acid in membrane phospholipids. Significantly increased 8-isoprostane levels were found in the SHR groups compared to the WKY groups with the same exposure for all groups as well as control groups. Our results are consistent with several studies describing elevation of 8-isoprostane level in biological samples in hypertension [63,64,65].

Correlations between markers of oxidative stress in rats of each genotype confirm the positive effect of plasma antioxidant capacity or antioxidant enzymes on oxidative damage to proteins and lipid peroxidation. When plasma antioxidant capacity is lower in normotensive rats, there is higher formation of products of oxidative damage to proteins—AOPP and protein carbonyls. The higher the levels of AOPP in plasma, the higher the levels of other markers of oxidative damage to proteins (protein-carbonyls) and lipids (lipoperoxides). There is also a positive correlation between AOPP and GPx activity, i.e., higher AOPP levels indicate increased oxidative stress in animals, which leads to the activation of GPx.

According to our results, the effects of USPIONs and psycho-emotional stress on the induction of oxidative stress depend on various conditions. Genotype can play a very important role. Of course, other factors such as time of exposure or dose can also impact this process.

## 5. Conclusions

**Figure 5:**
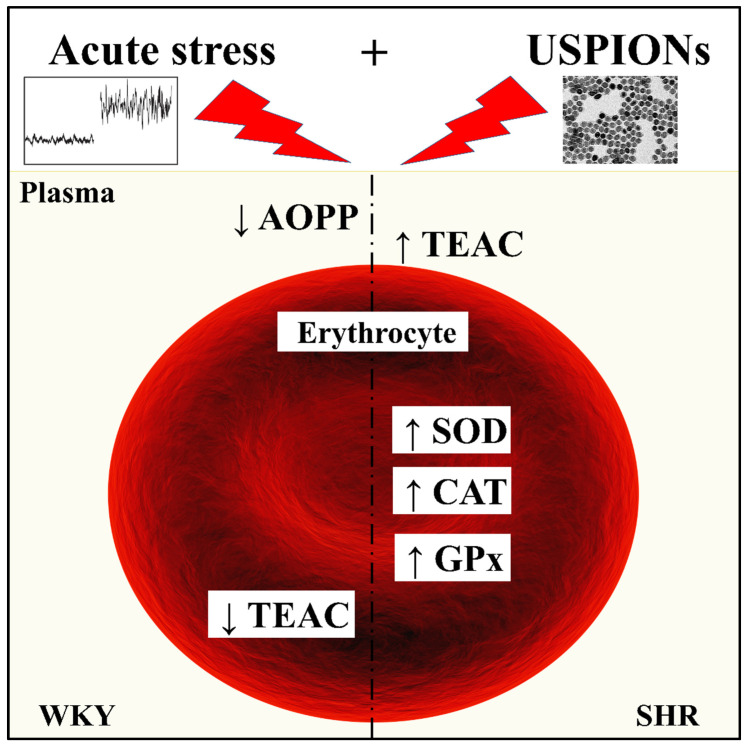
Highlights. AOPP—advanced oxidation protein products; TEAC—Trolox Equivalent Antioxidant Capacity; SOD—superoxide dismutase; GPx—glutathione peroxidase; CAT—catalase; WKY—Wistar–Kyoto rats; SHR—spontaneously hypertensive rats.

Our results show (Figure 5) significant differences in plasma antioxidant status, the activities of antioxidant enzymes and markers of oxidative damage to proteins and lipids in SHR under acute stress and during administration of USPIONs, or their combination. We found that SHR exposed to USPIONs in combination with acute stress, despite the elevated activities of antioxidant enzymes, experience increased oxidative damage to lipids and proteins determined in plasma compared to normotensive rats (WKY). Thus, our data point to the fact that caution should be taken when hypertensive subjects are treated with USPIONs, especially in stress conditions, as they seem to be more vulnerable to oxidative stress produced by iron oxide nanoparticles.

## Figures and Tables

**Table 1:** Significant correlations between different parameters of oxidative stress in normotensive (WKY) and hypertensive (SHR) rats.

Genotype	Parameter	Parameter	*n*	*p*	r
WKY	AOPP	TEAC-PL	23	0.005	−0.568
	AOPP	LPx	23	0.038	0.435
	AOPP	Carb-P	23	0.047	0.418
	AOPP	GPx	23	0.018	0.488
	Carb-P	TEAC-PL	23	0.010	−0.525
	Carb-P	GPx	23	0.010	0.525
	GPx	TEAC-PL	23	0.0001	−0.716
SHR	AOPP	LPx	28	0.001	0.602
	AOPP	Iso-P	28	0.011	0.474
	SOD	GPx	28	0.0001	0.742

Correlation is significant at the 0.05 level (2 tailed). AOPP—advanced oxidation protein products, Carb-P—protein carbonyls, LPx—lipoperoxides, GPx—glutathione peroxidase, TEAC-PL—antioxidant capacity in plasma, IsoP—8-isoprostanes, and SOD—superoxide dismutase.

## Data Availability

The data presented in this study are available in this article.
